# Physiological and Genomic Characterization of a Hyperthermophilic Archaeon *Archaeoglobus neptunius* sp. nov. Isolated From a Deep-Sea Hydrothermal Vent Warrants the Reclassification of the Genus *Archaeoglobus*

**DOI:** 10.3389/fmicb.2021.679245

**Published:** 2021-07-16

**Authors:** Galina Slobodkina, Maxime Allioux, Alexander Merkel, Marie-Anne Cambon-Bonavita, Karine Alain, Mohamed Jebbar, Alexander Slobodkin

**Affiliations:** ^1^Winogradsky Institute of Microbiology, Federal Research Center of Biotechnology of the Russian Academy of Sciences, Moscow, Russia; ^2^Laboratoire de Microbiologie des Environnements Extrêmes LM2E, Univ Brest, CNRS, IFREMER, IRP 1211 MicrobSea, UMR 6197, Plouzané, France

**Keywords:** sulfate reduction, chemolithoautotroph, anaerobe, hyperthermophile, *Archaea*

## Abstract

Hyperthermophilic archaea of the genus *Archaeoglobus* are the subject of many fundamental and biotechnological researches. Despite their significance, the class *Archaeoglobi* is currently represented by only eight species obtained as axenic cultures and taxonomically characterized. Here, we report the isolation and characterization of a new species of *Archaeoglobus* from a deep-sea hydrothermal vent (Mid-Atlantic Ridge, TAG) for which the name *Archaeoglobus neptunius* sp. nov. is proposed. The type strain is SE56^T^ (=DSM 110954^T^ = VKM B-3474^T^). The cells of the novel isolate are motile irregular cocci growing at 50–85°C, pH 5.5–7.5, and NaCl concentrations of 1.5–4.5% (w/v). Strain SE56^T^ grows lithoautotrophically with H_2_ as an electron donor, sulfite or thiosulfate as an electron acceptor, and CO_2_/HCO_3_^−^ as a carbon source. It is also capable of chemoorganotrophic growth by reduction of sulfate, sulfite, or thiosulfate. The genome of the new isolate consists of a 2,115,826 bp chromosome with an overall G + C content of 46.0 mol%. The whole-genome annotation confirms the key metabolic features of the novel isolate demonstrated experimentally. Genome contains a complete set of genes involved in CO_2_ fixation *via* reductive acetyl-CoA pathway, gluconeogenesis, hydrogen and fatty acids oxidation, sulfate reduction, and flagellar motility. The phylogenomic reconstruction based on 122 conserved single-copy archaeal proteins supported by average nucleotide identity (ANI), average amino acid identity (AAI), and alignment fraction (AF) values, indicates a polyphyletic origin of the species currently included into the genus *Archaeoglobus*, warranting its reclassification.

## Introduction

Over several decades since their discovery, thermophilic archaea have been the subject of an increasing number of studies related to microbial ecology, biogeochemistry, origin of life, and evolution of the biosphere (Offre et al., 2013). It was long thought that hyperthermophilic archaea represented the deepest and shortest phylogenetic branches of the tree of life and could be considered the first living organisms on Earth since most of them have a chemolithoautotrophic mode of nutrition (Stetter, 2006; Martin and Sousa, 2016; Weiss et al., 2016), but this hypothesis is now being questioned. Indeed, numerous archaeal lineages, with new complete genomes or high-quality *metagenomes-assembled genomes* (MAGs) and *single-amplified genomes* (SAGs), have been discovered thanks to remarkable advances in sequencing techniques and in data processing capabilities, allowing the construction of robust phylogenetic trees based on several genetic markers, which demonstrate that the root is far from being resolved (Baker et al., 2020). Dissimilatory sulfate reduction is a microbial process with significant ecological and biogeochemical implication (Rabus et al., 2015). It is mainly related to the marine environments because of the high sulfate concentrations. In the marine ecosystems with temperatures above 80°C, the only conclusively proven actors of this process are the representatives of the genus *Archaeoglobus* that was established by Stetter et al. (1987) and Stetter (1988). The type strain, *A. fulgidus* VC-16^T^, is one of the best studied *Archaea*, as it was the first described hyperthermophilic sulfate-reducing archaea, and one of the first archaea whose genome has been sequenced (Stetter, 1988; Klenk et al., 1997). In addition to physiological properties given in the original description, new metabolic features were found based on genome sequencing data analysis, e.g., growth coupled with carbon monoxide oxidation and sulfate reduction or acetogenesis (Henstra et al., 2007); the ability for anaerobic oxidation of fatty acids, *n*-alkenes, and *n*-alkanes (Khelifi et al., 2010, 2014); and growth with (per)chlorate reduction combining biotic and abiotic reactions (Liebensteiner et al., 2013). Along with the type strain, *A. fulgidus* 7324 has been isolated from North Sea oilfield waters and its genome has been sequenced (Beeder et al., 1994; Birkeland et al., 2017).

*Archaeoglobus fulgidus* became a source and a model organism for the study of structure and function of many thermostable enzymes. A good example is *A. fulgidus* ferritin (AfFt) whose unique structure and properties make it useful in clinical therapy (Palombarini et al., 2020). *Archaeoglobus fulgidus* is also a promising candidate to clean up oil-contaminated environments at low cost and with high efficiency due to its high resistance to anaerobic conditions, high temperatures, and high salts concentrations, and its ability to degrade alkanes (Park and Park, 2018).

At the time of writing, the genus *Archaeoglobus* includes five species with validly published names: *A. fulgidus* (Stetter, 1988), *A. profundus* (Burggraf et al., 1990), *A. veneficus* (Huber et al., 1997), *A. infectus* (Mori et al., 2008). and *A. sulfaticallidus* (Steinsbu et al., 2010). Together with related genera, *Geoglobus* and *Ferroglobus*, they compose the family *Archaeoglobaceae* belonging to the order *Archaeoglobales*, class *Archaeoglobi* (Parte et al., 2020; http://www.bacterio.net/index.html). It is notable that class *Archaeoglobi* is represented by only eight species obtained as axenic cultures and taxonomically characterized in detail. All known representatives of *Archaeoglobi* are strict anaerobes and hyperthermophiles isolated from marine hydrothermal systems and off-shore oil reservoirs. With the exception of *A. infectus* and *A. profundus*, all species of the order *Archaeoglobales* are capable of chemolithoautotrophic growth. Members of this order differ significantly in terms of the electron acceptors they use. All known species of the genus *Archaeoglobus* are able to grow by reduction of sulfite and thiosulfate. *Archaeoglobus fulgidus*, *A. profundus*, and *A. sulfaticallidus* can also reduce sulfate with organic carbon source, but only *A. sulfaticallidus* is capable of lithoautotrophic growth with sulfate as a terminal electron acceptor. Species of the genus *Geoglobus* are obligate iron-reducers (Kashefi et al., 2002; Slobodkina et al., 2009). The only representative of the genus *Ferroglobus*, *F. placidus,* can use nitrate, thiosulfate, and Fe^3+^ as electron acceptors (Hafenbradl et al., 1996; Tor et al., 2001).

Over the last decade, genomic data have increased exponentially and revealed many phylogenetic inconsistencies that require revision and reclassification of existing prokaryotic taxonomy and nomenclature. These taxonomic issues were recently addressed by proposing a standardized taxonomy for *Bacteria* and *Archaea* referred to as the Genome Taxonomy DataBase (GTDB; Parks et al., 2018; https://gtdb.ecogenomic.org/), based on average nucleotide criteria to delineate species and on sets of protein markers to infer a taxonomic position and define genomic clusters. *Archaea* were recognized as a separate domain in 1977 on the basis of small subunit (SSU) ribosomal RNA (rRNA) gene sequences analysis (Woese and Fox, 1977) and this phylogenetic marker was used for their classification afterward. Therefore, compared to *Bacteria*, which classification has long been relied on phenotypic properties, the *Archaea* are less affected by historical misclassifications (Zuo et al., 2015). The *Archaeoglobus* species were classified into the genus based mostly on phylogenetic analyses of 16S rRNA gene sequences. According to the GTDB, the genus *Archaeoglobus* encompasses four distinct clusters of genomic sequences. In this paper, we describe a novel thermophilic and facultative lithoautotrophic strain SE56^T^, belonging to the genus *Archaeoglobus*. To further understand the physiological features and genome characteristics of this novel species, the whole-genome of strain SE56^T^ was sequenced and analyzed. An advanced phylogenomic reconstruction based on 122 conserved single-copy archaeal protein markers and supported by *Average Nucleotide Identity* (ANI), *Alignment Fraction* (AF), and *Average Amino Acid Identity* (AAI) values clearly indicated a polyphyletic origin of the species currently affiliated to the genus *Archaeoglobus*.

## Materials and Methods

### Origin of the Strain

Strain SE56^T^ was isolated from a hydrothermal diffuser fragment (from the sample BIC2-PL1917-11-PBT1-02) collected in 2018 during the BICOSE 2 oceanographic cruise,^1^ using the manned operated submersible Nautile, at the TAG vent field (26°13'69"N 44°82'61"W, 3,625 m water depth) on the Mid-Atlantic Ridge. Onboard, the samples were immediately subsampled under sterile conditions. Small rock fragments were placed into 50 ml glass flasks with *in situ* seawater under N_2_ flow, closed with a rubber stopper and aluminum cap, and were stored at 4°C.

### Media and Cultivation

The medium for enrichment and isolation contained (per liter distilled water): 0.33 g NH_4_Cl, 0.33 g KCl, 0.33 g CaCl_2_∙6H_2_O, 0.33 g KH_2_PO_4_, 18.0 g NaCl, 4.33 g MgCl_2_∙6H_2_O, 2.0 g NaHCO_3_, 0.001 g resazurin, 0.5 g Na_2_S·9H_2_O, 1 ml trace element solution (Slobodkin et al., 2012) and 1 ml vitamin solution (Wolin et al., 1963). The isolation medium had a pH of 6.5–6.8 (measured at 25°C). The medium was dispensed in 10 ml portions into 17 ml Hungate tubes and heat-sterilized at 121°C for 30 min; the gas phase consisted of H_2_/CO_2_ (20: 80 v/v). Sodium sulfite from a sterile anoxic stock solution was added as an electron acceptor to a final concentration of 5 mM. All transfers and sampling of cultures were performed with syringes. All incubations were at 80°C unless otherwise noted.

### Phenotypic Characterization

Cell growth was determined after acridine orange staining, by direct cell counting using a phase-contrast microscope (Olympus CX-41, Olympus Corp., Japan). The morphological properties of strain SE56^T^ were observed using light microscopy and transmission electron microscopy (JEM-100, JEOL, Japan). For Gram-staining cells from exponential and stationary phases of growth were used (Gerhardt et al., 1994). Determination of temperature, pH, and salinity ranges for growth was carried out as described previously (Slobodkina et al., 2016) in the same medium used for isolation, with pyruvate (10 mM) instead of molecular hydrogen. The influence of temperature on growth of strain SE56^T^ was determined in the range 40–95°C with an interval of 5°C. The effect of pH on growth was investigated at 80°C in the range 4.0–9.5 with a 0.5 pH unit interval. The salt requirement was tested at 80°C and pH 6.5 in the range 0–7% NaCl (w/v) with an interval of 0.5% (w/v). All experiments were performed in duplicate, whereas testing the effect of pH on growth was performed in triplicate. Soluble electron donors and acceptors were added from sterile anoxic stock solutions before inoculation. Elemental sulfur was added directly into each test tube with liquid medium prior to tindallisation. Fe(III) was provided in the form of amorphous iron(III) oxide (ferrihydrite) at about 90 mmol Fe(III) L^−1^. The ferrihydrite was synthesized by titrating a solution of FeCl_3_, with 10% (w/v) NaOH to pH 9.0. The effect of O_2_ on growth of strain SE56^T^ was tested in 60 ml flasks containing 10 ml of the medium The flasks were sealed with a rubber stopper and aluminum screw cap. To check microaerobic growth, anoxic medium with pyruvate (10 mM) and without an electron acceptor, reducing agent, and resazurin was used. The headspace of the flask was filled with CO_2_ (100%). Various amounts of sterile air were injected by syringes in the headspace to obtain oxygen concentrations of 0.5, 2.0, or 5.0% (v/v). The ability of the strain to aerobic growth was determined in the same medium prepared in air. The strain was regarded as utilizing the added electron acceptors and donors if growth was sustained after at least three subsequent transfers into fresh medium.

### Genome Sequencing, Assembly, and Analysis

For genomic DNA extraction, the strain was cultivated at 80°C with pyruvate (10 mM) and sodium sulfite (5 mM) as electron donor and acceptor, respectively. Cells were harvested in the late exponential phase of growth by centrifugation at 4°C (15,000 × *g*, 20 min). The DNA was extracted using a FastDNA™ Spin Kit (MP Biomedicals, United States) according to the manufacturer’s instructions. The whole genome was sequenced using the Illumina nanoMiSeq technology (Fasteris, Switzerland; 2 × 150 paired-reads, Nano V2 chemistry). Clean reads from short-reads sequencing were assembled and circularized using SPAdes v1.13.1 assembly pipeline (Bankevich et al., 2012; https://github.com/ablab/spades). Average coverage was calculated using BBtools (BBMap – Bushnell B., v38.70 – sourceforge.net/projects/bbmap/). Contamination and completeness were assessed by СheckM v1.1.2 (Parks et al., 2015; https://github.com/Ecogenomics/CheckM). Gene search and annotation were performed by means of the Rapid Annotation using Subsystem Technology (RAST)/SEED v2.0 pipeline (Overbeek et al., 2014; https://rast.nmpdr.org/), the Integrated Microbial genomes (IMGs)/M v.5.0 analysis system (Chen et al., 2019; https://img.jgi.doe.gov/), and National Center of Biotechnology Information (NCBI) integrated prokaryotic genome annotation pipeline (PAGP; Tatusova et al., 2016). Identification and classification of the clustered regularly interspaced short palindromic repeats (CRISPR)-Cas system was performed by the CRISPRCas Finder web server (Couvin et al., 2018; https://crispr.i2bc.paris-saclay.fr/Server/). The prediction of laterally transferred gene clusters [genomic islands (GI)] was performed with the IslandViewer4 web server (Bertelli et al., 2017; http://www.pathogenomics.sfu.ca/islandviewer/). Genome and gene clusters visualization was made with the CGView program (Grant and Stothard, 2008; https://paulstothard.github.io/cgview/) and Gene Graphics web application (Harrison et al., 2018; https://katlabs.cc/genegraphics/app). Analysis of the clusters of orthologous genes (COG) functional categories was performed with the eggNOG-Mapper v2 (Huerta-Cepas et al., 2017; http://eggnog-mapper.embl.de/). Hydrogenase classification has been checked using the HydDB webtool (Søndergaard et al., 2016; https://services.birc.au.dk/hyddb/).

### Phylogenetic and Phylogenomic Analyses

For phylogenetic analysis, the 16S rRNA gene sequence of the isolate (retrieved from the whole-genome sequencing) was compared with other sequences in GenBank (Benson et al., 1999) using the BLAST program (Altschul et al., 1990; http://www.ncbi.nlm.nih.gov/BLAST/) and by means of the EzBio-Cloud server (Yoon et al., 2017; https://www.ezbiocloud.net/) to identify its closest relatives. Alignment with a representative set of related 16S rRNA gene sequences from the GenBank database was carried out with the CLUSTALW program implemented in the MEGA software package v7.0 (Kumar et al., 2016). Evolutionary analysis and phylogenetic tree reconstruction used the Maximum-Likelihood algorithm based on the Tamura–Nei model and the Neighbor-Joining methods (Saitou and Nei, 1987; Tamura and Nei, 1993) provided by MEGA v7.0. ANI were calculated using three methods: (i) OrthoANIu, the orthologous ANI algorithm using the USEARCH program (Edgar, 2010) provided by the EzBioCloud ANI calculator^2^; (ii) ANIb, the algorithm using BLASTN (Goris et al., 2007) provided by JSpeciesWS Online Service (Richter et al., 2016; http://jspecies.ribohost.com/jspeciesws/#analyse); and (iii) gANI obtained by the Microbial Species Identifier (MiSI) method (Varghese et al., 2015) using ANIcalculator implemented in the IMG/M system.^3^ The alignment fraction (AF) values were also obtained *via* the Pairwise ANI tool implemented in the IMG/M online service. Digital DNA–DNA hybridizations (dDDH) were performed using the genome-to-genome distance (GGDC) method with the GGDC 2.0 blast+ model provided by the Genome-to-Genome Distance Calculator (Meier-Kolthoff et al., 2013; https://www.dsmz.de/). The AAI between the selected genomes was calculated using the aai.rb script from the enveomics collection.^4^ The list of 122 archaeal marker genes used for phylogenetic inference was taken from the GTDB (Parks et al., 2018). These marker genes were extracted from genomes using GTDB-Tk v1.3.0 (Chaumeil et al., 2019; https://github.com/Ecogenomics/GTDBTk), aligned using mafft v7.475 (Katoh and Standley, 2013), trimmed using trimAl 1.2 (Capella-Gutiérrez et al., 2009), and concatenated. The phylogenomic tree was built using PhyML v3.3.2 (Guindon et al., 2010) and the Bayesian like transformation of approximate likelihood-ratio test for branches (Anisimova et al., 2011). LG was selected as the best substitution model by the SMS algorithm (Le and Gascuel, 2008; Lefort et al., 2017).

## Results and Discussion

### Isolation of the Strain SE56^T^

An enrichment culture was initiated by inoculation of the mixture of small rock fragments and sea water [at 10% (w/v)] into anoxically prepared, bicarbonate-buffered, sterile liquid medium with molecular hydrogen as an electron donor, sulfite as an electron acceptor, and CO_2_/HCO_3_^−^ as a carbon source. After incubation in the dark for 10 days at 80°C, growth of irregular-shaped cells was observed. After three subsequent transfers and following serial 10-fold dilutions in the same medium, only one morphological type was observed in the highest growth-positive dilution (10^−6^). A pure culture of strain SE56^T^ was obtained by performing multiple dilution-to extinction series in the same medium. Purity of the isolate was confirmed by routine microscopic examination on various media, and sequencing of the 16S rRNA gene and genome.

### 16S rRNA Gene Phylogenetic Analysis

**Figure 1:**
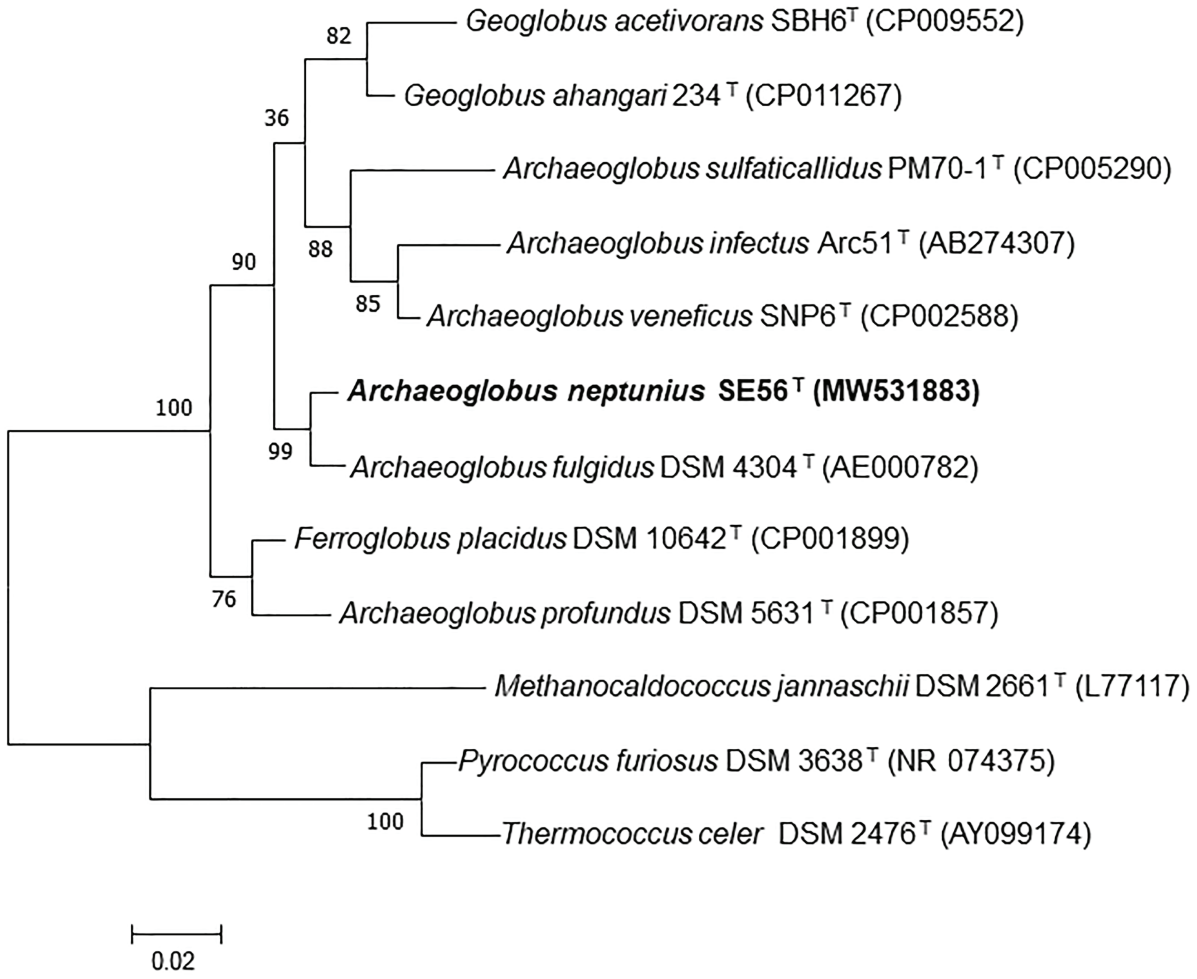
Maximum-likelihood phylogenetic tree based on 16S rRNA gene sequences showing the position of the strain SE56^T^ among the other members of the class *Archaeoglobi*. Bootstrap values based on 1,000 replicates are shown at branch nodes. *Methanocaldococcus jannaschii* DSM 2661^T^, *Pyrococcus furiosus* DSM 3638^T^, and *Thermococcus celer* DSM 2476^T^ were used as outgroup. Bar, 0.02 substitutions per nucleotide position.

Phylogenetic analysis based on comparison of 16S rRNA gene sequences showed that the strain SE56^T^ belonged to the genus *Archaeoglobus* (Figure 1; Supplementary Figure S1) and had the highest sequence similarity to *A fulgidus* VC-16^T^ (98.6%). Sequence similarities between the 16S rRNA gene sequence of the isolate and those of other representatives in the order *Archaeoglobales* were 96.3–94.4%.

### Cell Morphology, Physiology, and Metabolism

**Figure 2:**
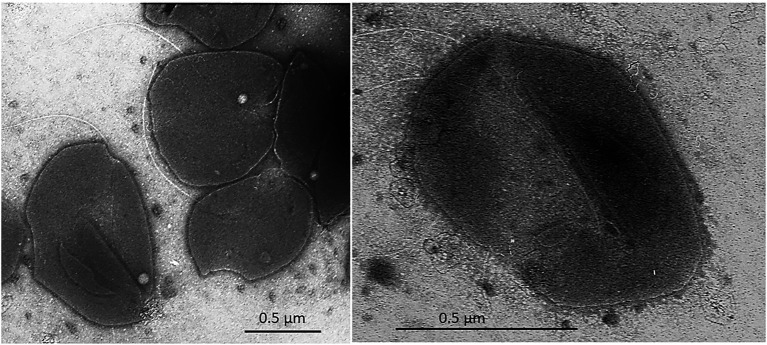
Transmission electron micrograph (TEM) of negatively stained cells of *Archaeoglobus neptunius* SE56^T^ sp. nov. shows an overall cell morphology. Bar, 0.5 μm.

Cells of strain SE56^T^ were motile irregular cocci (approximately 0.6–0.8 μm in diameter), usually occurring singly (Figure 2). Cells stained Gram-negative and showed blue-green fluorescence when exposed to UV under a fluorescence microscope.

The temperature range for the growth of strain SE56^T^ was 50–85°C, with an optimum at 80°C. No growth was detected at 90°C or above, neither at 45°C or below, after 3 weeks of incubation. The pH range for growth was 5.5–7.5, with an optimum at pH 6.5. No growth was observed at pH 5.0 or below or at pH 8.0 or above. Growth of the isolate was observed at NaCl concentrations ranging from 1.5 to 4.5% (w/v), with an optimum at 2.0–2.5% (w/v). No growth was evident neither at 5.0% NaCl (w/v) or above, nor at 1.0% NaCl (w/v) and below (Supplementary Figure S2). The doubling time under optimal conditions was about 2 h. Yeast extract was not necessary for growth and did not stimulate it.

Growth with potential organic substrates was tested with sulfite as a terminal electron acceptor. The best growth (final cell density 2–4∙10^8^ cells ml^−1^) was observed with pyruvate (10 mM), but growth was not observed after 7 days of incubation in the same conditions with the addition of elemental sulfur (5 g L^−1^). Acetate propionate, butyrate, fumarate, succinate (10 mM each), stearate (1 mM), peptone, tryptone, and yeast extract (2 g L^−1^ each) also sustained growth of the isolate (final cell density 5–9∙10^7^ cells ml^−1^). No growth was observed with formate, methanol, ethanol, isopropanol, glycerol, citrate, lactate, malate (10 mM each), fructose, glucose, sucrose, maltose, xylose, starch, and alginate (2 g L^−1^ each) during 4 weeks of incubation under optimal growth conditions.

Potential electron acceptors were tested with molecular hydrogen [H_2_/CO_2_ (20:80, v/v)], pyruvate (10 mM), or in simultaneous presence of hydrogen and pyruvate as energy and/or carbon sources. Strain SE56^T^ could grow lithoautotrophically with molecular hydrogen as an electron donor, sulfite (5 mM) or thiosulfate (15 mM) as a terminal electron acceptor, and CO_2_/HCO_3_^−^ as carbon source. Under lithoautrotrophic conditions with sulfate (15 mM) as a terminal electron acceptor, no growth was observed. Growth with sulfate (15 mM), sulfite (5 mM), or thiosulfate (15 mM) was observed with pyruvate (10 mM) or with hydrogen combined with pyruvate. Elemental sulfur (5 g L^−1^), fumarate, nitrate (10 mM each), nitrite (2.5 mM), chlorate, perchlorate (5 mM each), and ferrihydrite [90 mmol Fe(III) L^−1^] did not support growth under any tested conditions. Strain SE56^T^ did not grow aerobically or when oxygen (0.5, 2.0, or 5.0%, v/v) was added to the gas phase. No growth was observed with pyruvate (10 mM), yeast extract, or peptone (2 g L^−1^ each) in the absence of an electron acceptor.

The novel isolate shares general features with the other members of the genus, such as morphology, growth conditions, capacity to reduce oxidized sulfur compounds, and inability to grow through iron or nitrate reduction. The differential characteristics of strain SE56^T^ and other species of the genus *Archaeoglobus* are summarized in Table 1.

**Table 1:** Differential characteristics of strain SE56^T^ and the type stains of the species of the genus *Archaeoglobus*.

	*A. neptunius* SE56^T^	*A. fulgidus* VC-16^T^	*A. profundus* AV18^T^	*A. veneficus* SNP6^T^	*A. infectus* Arc51^T^	*A. sulfaticallidus* PM70-1^T^
Location of hydrothermal vent, water depth	Mid-Atlantic Ridge, TAG vent field, 3,625 m	Shallow-sea hydrothermal vent, Volcano, Italy	Guaymas Basin, Mexico, 2,000 m	Mid-Atlantic Ridge, “Snake Pit” site, 3,500 m	Pacific Ocean, Suiyo Seamount, Izu-Bonin Arc, 1,380 m	Pacific Ocean, Juan de Fuca Ridge, 2,658 m
Cell size, μm	0.6–0.8	0.1–1.0	1.3	0.5–1.2	0.5–1.0	0.4–2.2
Growth conditions						
Temperature (opt; °C)	50–85 (80)	60–95 (83)	65–90 (82)	65–85 (80)	60–75 (75)	60–80 (75)
pH (opt)	5.5–7.5 (6.5)	5.5–7.5 (ND)	4.5–7.5 (6.0)	6.5–8.0 (7.0)	6.5–7.0 (6.5)	6.3–7.6 (7.0)
NaCl (opt) % (w/v)	1.5–4.5 (2.0–2.5)	ND	0.9–3.6 (1.8)	0.5–4.0 (2.0)	1.0–4.0 (3.0)	0.5–3.5 (2.0)
Chemolithoautotrophic growth	+	+	−	+	−	+
SO_4_^2−^ reduction	+	+	+	−	−	+
Electron donors						
Formate	−	+	−	+	−	−
Butyrate	+	+	−	ND	−	−
Pyruvate	+	+	−	+	−	+
Lactate	−	+	−	−	−	+
Fumarate	+	−	−	w	−	−
Yeast extract	+	+	−	+	−	−

Data for reference strains were taken from Stetter, 1988; Burggraf et al., 1990; Huber et al., 1997; Mori et al., 2008 and Steinsbu et al., 2010 (*Archaeoglobus fulgidus* VC-16^T^, *Archaeoglobus profundus* AV18^T^, *Achaeoglobus veneficus* SNP6^T^, *Archaeoglobus infectus* Arc51^T^ and *Archaeoglobus sulfaticallidus* PM70-1^T^, respectively). All strains were isolated from marine hydrothermal vents. +, Positive; (w), weakly positive; −, negative; and ND, not determined.

### Genome Statistics

**Figure 3:**
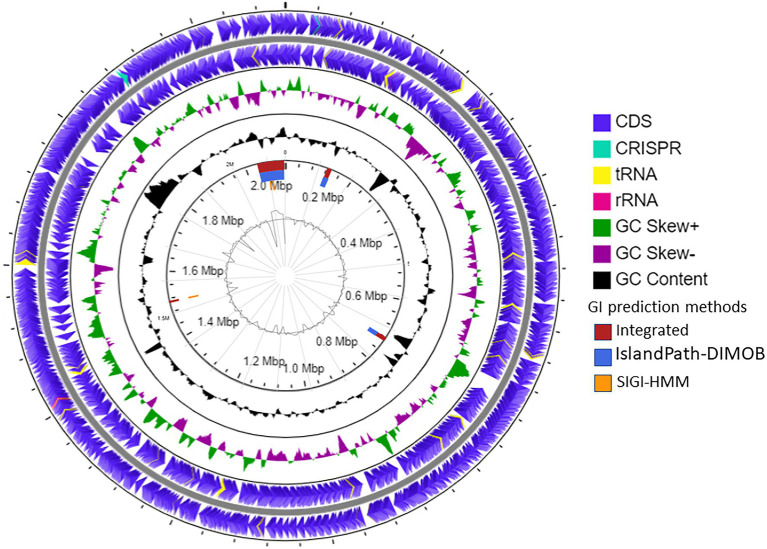
Schematic representation of the genome of *A. neptunius* SE56^T^. Labeling from the outside to the center is as follows: circle 1, genes on the forward strand; circle 2, genes on reverse strand [tRNAs yellow, ribosomal RNAs (rRNAs) lilac pink, and clustered regularly interspaced short palindromic repeats (CRISPR) cyan blue]; circle 3, G + C skew; circle 4, G + C content; circle 5, genomic islands shown as red, blue, and orange rectangles attributed, respectively, to integrated, IslandPath-DIMOB and SIGI-HMM prediction genomic islands methods; and circle 6, IslandViewer4 automatic calculation of the G + C content.

The draft genome of strain SE56^T^ was assembled into 32 contigs with genome size of 2,115,826 bp and a N_50_ value of 138,848 bp. Final assembly coverage was 153.537×. Based on CheckM analysis of single copy-core genes, the estimate of genome completeness was 100% and the estimate of contamination was 0%. The genomic DNA G + C content was 46.0 mol%. Annotation with prokaryotic genome annotation pipeline (PGAP) resulted in prediction of 2,471 genes, 2,386 of which are protein-coding sequences (CDSs) that cover about 96.5% of the entire genome. Genome also contained one copy of 5, 16, and 23S rRNA genes and 47 tRNA genes for all 20 standard amino acids. Two CRISPR loci were found (Figure 3).

Majority of the CDSs (72.3%) could be assigned to at least one COG group. The main predicted COG categories (encompassing more than 100 CDSs) were energy production and conversion (11.73%); amino acid transport and metabolism (8.60%); translation, ribosomal structure, and biogenesis (8.60%); transcription (6.73%); lipid transport and metabolism (5.87%); and coenzyme transport and metabolism (5.72%) and replication, recombination, and repair (5.36%).

The prediction of laterally transferred genes showed that the genome of strain SE56^T^ possesses four GI with a total length of 112.5 kb (Figure 3). Few genes were found encoding for enzymes related to archaeal chromosome replication, segregation, and cell division, such as ribonucleotide reductase, DNA helicase, transcription factor B, chromosome partitioning protein ParA, and cell division protein FtsH. Genes for 5'-nucleotidase SurE, AMP phosphorylase, and ribose-5-phosphate isomerase A were present as well, while the majority of CDSs located on the genomic islands encoded for hypothetical proteins (Supplementary Table S1).

### Central Carbon Metabolism

Strain SE56^T^ is capable of autotrophic growth using CO_2_ as a carbon source. Like all *Archaeoglobi* species except *A. profundus*, strain SE56^T^ has the complete set of genes for the CO_2_ fixation *via* reductive acetyl-CoA (Wood-Ljungdahl) pathway (Table 2; Klenk et al., 1997; von Jan et al., 2010; Anderson et al., 2011; Stokke et al., 2013; Manzella et al., 2015; Mardanov et al., 2015). The genome also contains a gene coding for the large subunit of ribulose-1,5-bisphosphate carboxylase/oxygenase (RubisCO, WP_202319886). This enzyme belongs to form III RubisCO, which does not participate in autotrophy in *Archaea* (Berg, 2011). Many other enzymes of the Calvin-Benson cycle are missing. The key enzymes of other known microbial carbon fixation pathways, namely, ATP-citrate lyase and citryl-CoA lyase (two variants of the reductive tricarboxylic acid cycle), malonyl-CoA reductase (3-hydroxypropionate bi-cycle), and most of enzymes participating in 3-hydroxypropionate/4-hydroxybutyrate and dicarboxylate/4-hydroxybutyrate cycles are also absent.

**Table 2:** Gene sets encoding various physiological properties in genomes of type strains of *Archaeoglobi*.

	CO_2_ fixation (W-L)	Gluconeogenesis (E-M)	Sulfate reduction	Fatty acids β-oxidation	Hydrogen oxidation	Flagella assembly	Chemotaxis
*A. neptunius* SE56^T^	C	C	C	C	+	+	+
*A. fulgidus* VC-16^T^	C	C	C	C	+	+	+
*A. profundus* AV 18^T^	INC	C	C	INC	+	+	+
*A. veneficus* SNP6^T^	C	C	C	INC	+	+	+
*A. sulfaticallidus* PM70-1^T^	C	C	C	INC	+	+	−
*G. ahangari* 234^T^	C	C	INC	C	+	+	−
*G. acetivorans* SBH6^T^	C	C	INC	C	+	+	+
*F. placidus* DSM 10642^T^	C	C	INC	C	+	+	+

W-L, Wood-Ljungdahl pathway; E-M, Embden-Meyerhof pathway; C, complete gene set for the pathway; INC, incomplete gene set for the pathway; +, genes present; and −, genes absent.

The genome of SE56^T^ contains most of the genes encoding the Embden-Meyerhof pathway enzymes (Table 2), with the exception of the enzymes that catalyze irreversible reactions of glycolysis, pyruvate kinase, and 6-phosphofructokinase. Instead, there are genes encoding fructose-bisphosphatase (WP_202319097) and phosphoenolpyruvate synthase (WP_202318379) that enable glucose production from pyruvate. Thus, the Embden-Meyerhof pathway in this organism operates in reverse direction, toward gluconeogenesis.

Although strain SE56^T^ lacks the key enzymes of the “standard” tricarboxylic acid cycle (TCA) cycle, aconitase and 2-oxoglutarate dehydrogenase complex, most of the genes involved in this cycle including citrate synthase (WP_202320798) are present. In this case, 2-oxoglutarate synthase may substitute the missing 2-oxoglutarate dehydrogenase. At least, two genes coding for 2-oxoglutarate synthase (*oorCBAD*) were found in the genome of strain SE56^T^. As well as the majority of *Euryarchaeota* whose genomes are sequenced, the genome of strain SE56^T^ does not encode orthologs of aconitase A or members of other aconitase families. A putative family of aconitases, aconitase X, has been proposed on the basis of comparative-genomic analysis (Makarova and Koonin, 2003). The predicted aconitase X consists of two proteins that function together as a TCA cycle enzyme catalyzing the citrate to isocitrate isomerization. Experimental validation of this prediction has not yet been received. Nevertheless, a complete oxidative TCA is declared to function in the cells of class *Archaeoglobi* members (*A. fulgidus*, *A. profundus*, *F. placidus*, *Geoglobus ahangari*, and *Geoglobus acetivorans*). In the genome of strain SE56^T^, two genes (WP_202319516 and WP_202319760) were found encoding proteins that have 70% similarity with the proteins of the putative aconitase X from *A. fulgidus* (AF2333 and AF0055). Alternatively, incomplete TCA cycle can be used in the isolate SE56^T^ to provide intermediates for anabolic pathways. Acetyl-CoA formed in Wood-Ljungdahl pathway or from acetate can be converted to pyruvate by a pyruvate synthase POR (*porBADG*, WP_202318910–WP_202318913). Pyruvate is converted to oxaloacetate in an irreversible reaction catalyzed by pyruvate carboxylase (*pycAB*, WP_202320749, and WP_202320510). Further formation of the necessary biosynthetic intermediates, in particular, succinyl-CoA and 2-oxoglutarate, is enabled by the presence of corresponding enzymes. This pathway has been experimentally shown in methanogenic archaea (Fuchs and Stupperich, 1978; Shieh and Whitman, 1987). Each of *Archaeoglobi*’s available genome contains homologs of all the genes involved in this incomplete TCA cycle.

The capacity of strain SE56^T^ to utilize propionate is confirmed by a number of genes putatively encoding enzymes involved in propionate oxidation *via* the methylmalonyl-CoA pathway. This includes glutaconate CoA-transferase (WP_202318740 and WP_202318741) and a short *mmc* cluster (WP_202319622–WP_202319626) consisting of propionyl-CoA carboxylase, methylmalonyl-CoA epimerase and methylmalonyl-CoA mutase. Several enzymes such as succinyl-CoA synthase (SCS; WP_202320107 and WP_202320108), succinate dehydrogenase (SDH; WP_202319710, WP_202319660-, and WP_202319663), and fumarate hydratase (FHT; WP_202319682 and WP_202319683) can participate in both propionate oxidation and TCA cycle. The NAD-dependent malic enzyme (*maeA*, WP_202320482), pyruvate ferredoxin oxidoreductase (*porBADG*, WP_202318910–WP_202318913), and/or formate acetyltransferase (WP_202319769) can be involved in final steps of propionate oxidation to acetyl-CoA.

The genome contains a large number of genes coding for fatty acid utilization enzymes *via* β-oxidation pathway (Table 2), including long-chain acyl-CoA synthetases, acyl-CoA dehydrogenases, enoyl-CoA hydratase/3-hydroxyacyl-CoA dehydrogenase, and acetyl-CoA acyltransferases. The genome also possesses 13 genes encoding acetyl-CoA C-acetyltransferase that are involved in fatty acid metabolism too, mainly in butyrate oxidation. This is consistent with the ability of strain SE56^T^ to utilize butyrate and stearate. *Archaeoglobus fulgidus*, as well as the species of the related genus *Geoglobus*, are known to use fatty acids as electron donors (Kashefi et al., 2002; Slobodkina et al., 2009; Khelifi et al., 2010). In the genomes of these organisms and in the genome of *Ferroglobus placidus*, a large number of genes encoding the core enzymes of fatty acid β-oxidation have been found (Klenk et al., 1997; Anderson et al., 2011; Manzella et al., 2015; Mardanov et al., 2015). In contrast, in the genome of *A. veneficus*, we found only one gene of long-chain acyl-CoA synthetase and two genes of acetyl-CoA acetyltransferase. The genome of *A. sulfaticallidus* which was reported as unable to utilize butyrate (Steinsbu et al., 2010), lacks genes of two of the key enzymes for β-oxidation: enoyl-CoA hydratase and 3-hydroxyacyl-CoA dehydrogenase. The same genes are missing in the genome of *A. profundus*, as previously reported (von Jan et al., 2010).

Our attempts to grow strain SE56^T^ with lactate as the electron donor and sulfite or sulfate as the electron acceptor were unsuccessful, although two known strains of *A. fulgidus* are capable of oxidizing lactate with sulfate. The genes for D-lactate and L-lactate dehydrogenases (*dld*, AF0394 and *lldD*, AF0807) were annotated in the genome of the type strain *A. fulgidus* (Klenk et al., 1997). The transcriptomic study proposed that lldD (AF0807) is part of a cluster and operates along with L-lactate permease (AF0806), monomeric dld (AF0808), and oligomeric LldEFG (AF0809−AF0811) lactate dehydrogenases (Hocking et al., 2014). This study also suggested to consider two more oxidoreductases (AF0507 and AF0867) as putative lactate dehydrogenases. In the genome of strain SE56^T^, we have found close homologs (62–78%) of proteins encoded by AF0394, AF0507, and AF0867 and the homologs of the subunits of LldEFG lactate dehydrogenase. Yet, the homologs of L-lactate permease and lactate dehydrogenases lldD and dld, encoded by the genes AF0806−AF0808 were absent. Thus, the reason for the absence of growth of strain SE56^T^ with lactate is unclear. This may be because the missing enzymes are essential for lactate metabolism, or because the conditions for growth of strain SE56^T^ with lactate are not yet identified.

### Hydrogen Oxidation

A cluster of genes encoding a membrane-bound hydrogen uptake [NiFe] hydrogenase (Vht), a cytoplasmic [NiFe] hydrogenase:heterodisulfide (Mvh:HdrABC) complex, and a set of hydrogenase maturation proteins (WP_202319836−WP_202319849) provides strain SE56^T^ the ability to utilize molecular hydrogen (Table 2). This cluster is syntenic to the one encoding the hydrogenases in *A. fulgidus* (AF1365−AF1381). Large catalytic subunits of Vht hydrogenase (vhtA) and Mvh hydrogenase (mvhA) of strain SE56^T^ have 83 and 63% of amino acids similarities with corresponding proteins in *A. fulgidus*. According to a model of hydrogen metabolism proposed for *A. fulgidus* (Hocking et al., 2014), Vht hydrogenase is required for energy conservation which is achieved by electron transfer from hydrogen to sulfite *via* DsrMK/HdrDE complexes, where a particular putative heterodisulfide reductase HdrDE (AF0755) may play an important role. We detected close homolog of this HdrDE (WP_202318846) with 75% amino acid similarity in strain SE56^T^. Thus, the same model of hydrogenotrophy might be operative in *A. neptunius* SE56^T^ cells.

### Reduction of Sulfate and Other Potential Electron Acceptors

Genome of strain SE56^T^ contains the complete set of genes involved in dissimilatory sulfate reduction (Table 2), including sulfate adenylyltransferase *sat* and adenylyl-sulfate reductase *aprAB* (WP_202320080 and WP_202320082–WP_202320083), manganese-dependent inorganic pyrophosphatase *ppaC* (WP_202318845), dissimilatory sulfite reductase *dsrABD* (WP_202320149–WP_202320151), and *dsrC* which is more distantly located (WP_202320007). Genes coding for the electron transfer complexes *dsrMKJOP* (WP_202318457–WP_202318460) and *QmoABC* (WP_202319400–WP_202319402) are also present. Growth through sulfate reduction is a distinctive characteristic among species of the genus *Archaeoglobus*. *Archaeoglobus veneficus* and *A. infectus* were reported to be incapable to grow with sulfate as an electron acceptor (Huber et al., 1997; Mori et al., 2008). Meanwhile, the genome of *A. veneficus* contains a complete set of genes for dissimilatory sulfate reduction (Table 2), and *A. infectus* has *aprA* and *dsrAB* genes. A number of microorganisms did not demonstrate the growth due to dissimilatory sulfate reduction, despite the presence of complete set of genes for this pathway in their genomes (Finster et al., 2013; Slobodkin and Slobodkina, 2019). The reason of this is unclear; probably, special cultivation conditions are required.

**Figure 4:**
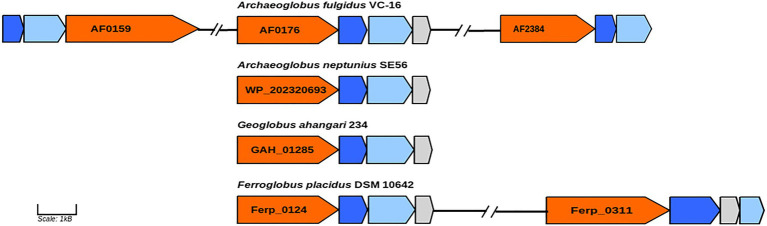
Genes of molybdopterin oxidoreductases probably involved in perchlorate and nitrate reduction. Orange, molybdopterin oxidoreductase molybdopterin binding subunit; blue, molybdopterin-containing oxidoreductase family iron–sulfur binding subunit; light blue, molybdopterin-containing oxidoreductase family membrane subunit; and gray: molybdenum cofactor insertion protein. GenBank accession numbers of catalytic subunits of each enzyme are shown.

In genome of the type species of the genus *Archaeoglobus*, several molybdopterin-binding oxidoreductases have been found, including a gene cluster (AF0174–AF0176) to which the function of a nitrate reductase has been assigned (Klenk et al., 1997; Richardson et al., 2001). Later, growth related to perchlorate reduction was demonstrated in this strain, and a two-stage model was proposed for this pathway (Liebensteiner et al., 2013). Based on genomic and proteomic evidence, it has been shown that perchlorate is reduced to chlorite by the molybdopterin-binding oxidoreductase encoded by the gene cluster AF0174–AF0176. Tetrathionate reductase (AF0157−AF0159) and an enzyme coded by the genes AF2384–AF2386 have been proposed as candidates for the second step of perchlorate reduction. Later transcriptome analysis showed upregulation of genes AF2384−AF2386 during growth with thiosulfate *vs* sulfate indicating that corresponding enzyme is a thiosulfate reductase (Hocking et al., 2014). In the genome of strain SE56^T^, we found a gene cluster (WP_202320693–WP_202320695) coding for proteins very similar to subunits of the perchlorate reductase of *A. fulgidus* (AF0174–AF0176). The amino acid similarity of the catalytic subunit (AF0176) to its homolog in strain SE56^T^ (WP_202320693) was 79%. However, the genome of SE56^T^ does not have close homologs to the genes of clusters AF0157–AF0159 and AF2384–AF2386, which could explain the inability of the strain to grow by (per)chlorate reduction. We did not find the close homologs to genes AF0157–AF0159 and AF2384–AF2386 in the genomes of the other *Archaeoglobi* representatives (Figure 4). Aside from the strain SE56^T^, we found close homologs to the catalytic subunit AF0176 (79% amino acids similarity) in the genomes of *Ferroglobus placidus* DSM 10642^T^ (Ferp_0124) and *Geoglobus ahangari* 234^T^ (GAH_01285). In both genomes, they are part of clusters coding for subunits of molybdopterin oxidoreductases. In *G. ahangari*, this cluster (GAH_01285–01288) has been previously designated as a nitrate reductase NarGHIJ (Manzella et al., 2015). The genome of *F. placidus* additionally contains another cluster, referenced Ferp_0311–0314, encoding three subunits (NarGHI) of the Nar-type respiratory nitrate reductase and the chaperone NarJ, which could explain why nitrate respiration could be demonstrated experimentally (Hafenbradl et al., 1996; Anderson et al., 2011). Our analysis showed that the alpha subunit of the nitrate reductase has the highest similarity with the nitrate reductase subunit alpha of *Nitrospirae* bacterium (71% amino acids similarity) and *Deltaproteobacteria* (67–68%). The other *Archaeoglobi* do not have close homologs to this enzyme and are unable to carry out dissimilatory nitrate reduction. The ability of *F. placidus* to reduce nitrate seems to have been acquired by horizontal transfer and to correspond to a quite recent evolutionary event.

### Motility and Chemotaxis

Strain SE56^T^ is motile and has a complete set of genes involved in proper assembly and function of flagella. The flagella apparatus operon *flaB1-2B1-1D/EGFHIJ* (WP_202320707–WP_202320713) found in the genome of strain SE56^T^ is characteristic of *Euryarcheota* (Albers and Jarrell, 2015). The gene encoding preflagellin peptidase (FlaK; WP_202320792), essential for the flagella assembly, is not in the same cluster in the assembly used for this study. The isolate also has the chemotaxis system to direct the movement towards more favorable location, including an operon *cheWFYBACDR* (WP_202319867–WP_202319871) and genes encoding homologs of several methyl-accepting chemotaxis (MCP) proteins. Unlike *A. fulgidus*, *A. profundus* and *A. veneficus*, flagella- and chemotaxis-coding genes do not cluster together in the strain SE56^T^. Among members of the genus *Archaeoglobus*, *A. profundus* and *A. sulfaticallidus* have been reported to be non-motile (Burggraf et al., 1990; Steinsbu et al., 2010). Our analysis of genome data of *A. sulfaticallidus* revealed the presence of a complete *fla* genes cluster as well as putative *flaK* gene (AGK60851–AGK60859; AGK61636). However, it lacks the chemotaxis system *che* genes that might explain the unobserved motility (Table 2). In contrast, *A. profundus* possesses both *fla* and *che* genes clusters which are virtually identical in content, order and orientation to corresponding genes of the motile *Archaeoglobi*. This discrepancy between genotype and phenotype has been noted earlier, and it was assumed that *A. profundus* can be motile in some specific conditions, or additional unknown factors are needed for motility (von Jan et al., 2010).

### Phylogenomic Analysis

The 16S rRNA gene-based phylogenetic analysis showed that strain SE56^T^ belongs to the genus *Archaeoglobus*. Further clarification of the taxonomic position of strain SE56^T^ was carried out using genome-based methods, ANI, AF, dDDH and AAI. At the time of writing, all genome sequences of *Archaeoglobales* type strains were available in public databases, with the exception of the type strain of *A. infectus*. The properties and statistics of genome sequences are presented in Table 3. The pairwise OrthoANIu value between genomes of strain SE56^T^ and its closest relative organism, *A. fulgidus* VC-16^T^ (NC_000917.1), was 73.49%. The OrthoANIu values with other available species were below 68.5%. Similar results were obtained by means of ANIb and MiSI algorithms (Table 3). The AF value for strain SE56^T^ and *A. fulgidus* VC-16^T^ was 0.62, whereas these values for strain SE56^T^ and other representatives of the order *Archaeoglobales* were 0.21–0.30 (Table 3). The predicted *in silico* DNA–DNA hybridization (dDDH) values between strain SE56^T^ and members of the order *Archaeoglobales* varied from 31.7% (*A. profundus* DSM 5631^T^) to 18.6% (*A. fulgidus* VC-16^T^). All these values are lower than the proposed threshold criteria for prokaryotic species delineation (95–96% ANI, 0.6–0.7 AF and 70% dDDH; Goris et al., 2007; Richter and Rosselló-Móra, 2009; Varghese et al., 2015).

**Table 3:** General genomic features and digital DNA–DNA hybridization (dDDH), average nucleotide identity (ANI), AAI, and alignment fraction (AF) values of strain *Archaeoglobus neptunius* SE56^T^ and the type strains of the related species.

	*A. neptunius* SE56^T^	*A. fulgidus* VC-16^T^	*A. profundus* AV18^T^	*A. veneficus* SNP6^T^	*A. sulfaticallidus* PM70-1^T^	*G. ahangari* 234^T^	*G. acetivorans* SBH6^T^	*F. placidus* DSM 10642^T^
Genome size (Mb)	2.12	2.18	1.56	1.9	2.08	1.77	1.86	2.2
G + C content (mol%)	46.0	48.6	42.0	47.0	43.2	53.1	46.8	44.1
CRISPR count	2	3	0	2	1	7	5	1
Number of genes	2,471	2,524	1846	2,180	2,304	2082	2,251	2,594
Protein coding genes	2,386	2,398	1764	2094	2,199	2001	2,177	2,487
Number of RNAs	52	51	52	51	56	51	53	54
dDDH (%)	100	18.6	31.7	26.8	23.3	22.2	20.4	23.7
ANIb (%)	100	72.58	67.06	67.94	68.0	67.43	67.8	67.19
OrthoANIu (%)	100	73.49	68.11	68.21	68.30	68.4	68.51	68.18
gANI (%)	100	74.03	68.52	68.92	69.26	69.13	69.14	68.62
AF	1.00	0.62	0.21	0.27	0.29	0.30	0.26	0.27
AAI (%)	100	74.88	57.63	59.23	59.0	58.91	57.86	57.84
Genbank ID	NZ_JAEKIW010000000	AE000782	CP001857	CP002588	CP005290	CP011267	CP009552	CP001899

**Figure 5:**
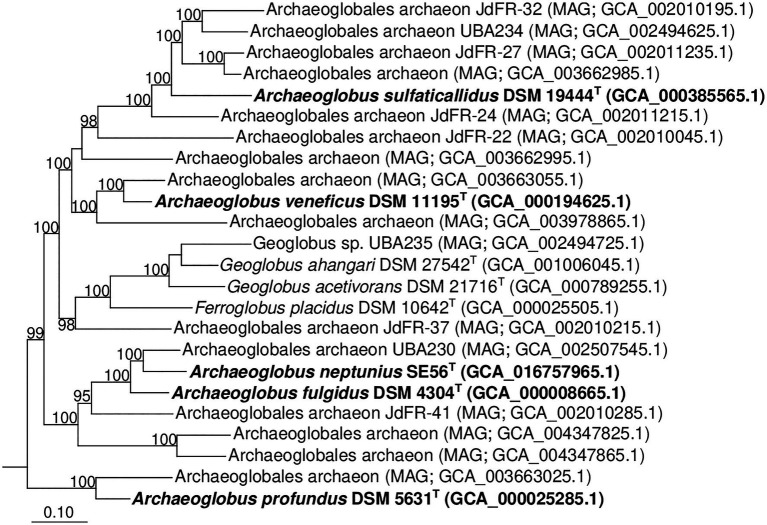
Phylogenomic placement of *A. neptunius* and other species of family *Archaeoglobaceae* based on concatenated partial amino acid sequences of 122 archaeal conservative proteins [Parks et al., 2018; taxonomic designations correspond with Genome Taxonomy DataBase (GTDB)]. The tree was built using the PhyML 3.0 program (Guindon et al., 2010). For rooting, the tree sequences from 62 genomes of type strains of type species of genera of “*Halobacteriota*” phylum (correspond with GTDB) were taken. All assemblages of *Archaeoglobaceae* family according to GTDB Release 05-RS95 are shown on the tree. Bootstrap values above 90% are shown at the nodes. Bar, 0.10 changes per position.

**Figure 6:**
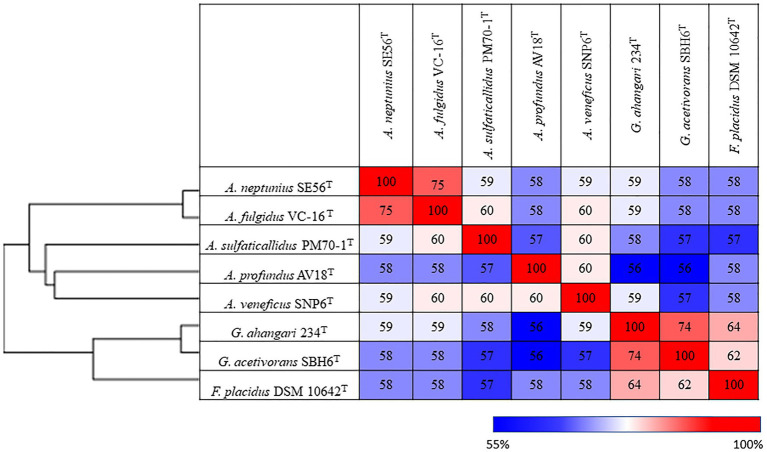
Pairwise average amino acid identity (AAI) determined for members of the class *Archaeglobi*.

Additionally, we studied the taxonomic position of strain SE56^T^ based on the alignment of a concatenated set of 122 single-copy partial amino acid sequences of conserved archaeal proteins. The phylogenomic reconstruction confirmed the position of strain SE56^T^ as a novel species within the genus *Archaeoglobus*. At the same time, it clearly indicated a polyphyletic origin of the species currently included into the genus *Archaeoglobus* (Figure 5). The AAI value between strain SE56^T^ and its closest relative, *A. fulgidus* VC-16^T^, was 74.88%. The AAI values between the other members of the genus *Archaeoglobus* were in the range of 57–60% (Figure 6). Corresponding ANI and AF values were in the range of 68–70% and 0.21–0.29, respectively (Supplementary Tables S2 and S3). At present, the AAI thresholds proposed for taxonomic delineation are 45–65% for the same family, 65–95% for the same genus and 95–100% for the same species (Konstantinidis et al., 2017). Other proposed genus demarcation criteria are 73.34–74.62% ANI and 0.308–0.354 AF (Barco et al., 2020). On this basis, strain SE56^T^ and *A. fulgidus* represent different species of one genus, while each of the other three known species of *Archaeoglobus* represents an individual genus, distinct from the first and distinct from each other. Among the other members of the class *Archaeoglobi*, only *G. ahangari* and *G. acetivorans* have AAI, ANI and AF values above the proposed genus threshold, that confirms the placement of these species into the same genus. Except strain SE56^T^ and *A. fulgidus*, as well as *Geoglobus* species, the AAI, ANI and AF values between all species of the *Archaeoglobi* class were in the range of 56–64%, 68–71%, and 0.21–0.39, respectively (Figure 6; Supplementary Tables S2 and S3), proving that they belong to different genera of the same family.

We therefore conclude, that, according to phylogenomic differentiation supported by ANI, AF and AAI values, the genus *Archaeoglobus* should be divided into four distinct genera. The genus name *Archaeoglobus* should be retained for the monophyletic group containing the type species, *A. fulgidus*. We propose to consider the strain SE56^T^ as a new species of the genus *Archaeoglobus*, *Archaeoglobus neptunius* sp. nov. All four proposed genera belong to the family *Archaeoglobaceae* and share the main morphological, physiological and metabolic features. Representatives of these genera are hyperthermophilic strict anaerobes with respiratory type of metabolism that use sulfur compounds as the terminal electron acceptors. All known species were isolated from marine ecosystems. In addition to the phylogenetic distance, each of the proposed genera has the distinctive phenotypic properties (Tables 1 and 2). The genus *Archaeoglobus sensu stricto* differs from the other three genera in its ability to utilize fatty acids provided by the presence of all the genes necessary for β-oxidation of fatty acids. A key distinguishing feature of the genus represented by *A. profundus* is the inability to grow lithoautotrophically caused by the incompleteness of Wood-Ljungdahl pathway. The genus represented by *A. sulfaticallidus* can use sulfate as an electron acceptor for lithoautotrophic growth with molecular hydrogen, whereas members of other genera can perform dissimilatory sulfate reduction only with organic carbon source. In addition, cells of *A. sulfaticallidus* are non-motile, lack genes encoding chemotaxis and have lower optimum growth temperature. The genus encompassing *A. veneficus* is distinguished by its inability to reduce sulfate although it has all the necessary genes. Further work, including the sequencing of the genome of *A. infectus* and core- and pan-genome analysis is required for the formal proposal of the reclassification of the genus *Archaeoglobus*.

### Description of *Archaeoglobus neptunius* sp. nov.

*Archaeoglobus neptunius* (nep.tu’ni.us L. masc. adj. *neptunius*, pertaining to Neptunius (Roman god of the sea), referring to the habitat of the microorganism).

Cells are Gram-negative irregular motile cocci of 0.6–0.8 μm in diameter usually occurring singly. Blue-green fluorescence under UV light. Strictly anaerobic. Growth occurs at 50–85°C (optimum at 80°C), pH 5.5–7.5 (optimum pH 6.5) and NaCl concentrations of 1.5–4.5% [w/v; optimum 2.0–2.5% (w/v) NaCl]. With sulfite or thiosulfate as an electron acceptor, it grows chemolithoautotrophically with molecular hydrogen as an electron donor and CO_2_/HCO_3_^−^ as a carbon source. Chemoorganotrophic growth on acetate, propionate, butyrate, fumarate, pyruvate, succinate, peptone, tryptone and yeast extract with sulfate, sulfite or thiosulfate as electron acceptor. Growth is inhibited by elemental sulfur. Formate, methanol, ethanol, iso-propanol, glycerol, lactate, malate, citrate, fructose, glucose, sucrose, maltose, xylose, starch and alginate are not utilized. Elemental sulfur, fumarate, nitrate, nitrite, chlorate, perchlorate and Fe(III) are not used as electron acceptors.

The type strain is SE56^T^ (=DSM 110954^T^ = VKM B-3474^T^), isolated from a deep-sea hydrothermal vent of the Mid-Atlantic Ridge (TAG vent field: 26°13'69"N 44°82'61"W, 3,625 m water depth). The genome consists of a 2,115,826 bp chromosome with an overall G + C content of 46.0 mol%.

GenBank/EMBL/DDBJ accession (16S rRNA gene): MW531883.

GenBank/EMBL/DDBJ accession (genome): NZ_JAEKIW010000000.

## Data Availability Statement

The Whole Genome Shotgun project has been deposited at DDBJ/ENA/GenBank under the accession JAEKIW000000000. The version described in this paper is version JAEKIW010000000. The BioSample data is available in the NCBI BioSample database (http://www.ncbi.nlm.nih.gov/biosample/) under the accession number SAMN17126929. The BioProject data is available in the NCBI BioProject database (https://www.ncbi.nlm.nih.gov/bioproject/) with BioProject ID: PRJNA686870. The 16S rRNA gene sequence of *A. neptunius* SE56T (retrieved from the whole-genome sequencing) has been deposited in GenBank/EMBL under accession number MW531883. The strain is available in the German Collection of Microorganisms and Cell Cultures (DSMZ) and All-Russian Collection of Microorganisms (VKM) under the accession numbers DSM 110954T and VKM B-3474T.

## Author Contributions

AS, GS, and MJ: conceptualization. GS, MA, AM, and KA: investigation. M-AC-B: resources. GS: writing – original draft preparation. AS, KA, AM, M-AC-B, and MA: writing – review and editing. GS and AM: visualization. AS and MJ: supervision and funding acquisition. All authors contributed to the article and approved the submitted version.

### Conflict of Interest

The authors declare that the research was conducted in the absence of any commercial or financial relationships that could be construed as a potential conflict of interest.

The reviewer TN declared a shared affiliation with one of the authors GS, AM, and AS to the handling editor at the time of the review.

## Footnotes

^1^http://dx.doi.org/10.17600/18000004

^2^https://www.ezbiocloud.net/tools/ani

^3^https://img.jgi.doe.gov/

^4^https://github.com/lmrodriguezr/enveomics

## Abbreviations

AAI: Average amino acid identity
AF: Alignment fraction
ANI: Average nucleotide identity
bp: Base pair
CDS: Coding DNA sequences
COGs: Clusters of orthologous groups
CRISPR: Clustered regularly interspaced short palindromic repeats
dDDH: digital DNA–DNA hybridization
DNA: deoxyribonucleic acid
EMBL: European Molecular Biology Laboratory
IMG/M: Integrated microbial genomes and microbiomes
GI: Genomic islands
GTDB: Genome taxonomy database
NCBI: National Center for Biotechnology Information
PGAP: Prokaryotic genome annotation pipeline
rRNA: Ribosomal ribonucleic acid
TCA: Tricarboxylic acid cycle
