# Supplementary material for: Physiological and Genomic Characterization of a Hyperthermophilic Archaeon Archaeoglobus neptunius sp. nov. Isolated From a Deep-Sea Hydrothermal Vent Warrants the Reclassification of the Genus Archaeoglobus
Source: Front Microbiol. 2021 Jul 16;12:679245. doi: 10.3389/fmicb.2021.679245 (PMC8322695; doi:10.3389/fmicb.2021.679245)
Supplement: Supplementary file 1 [file Presentation_1.PPTX]

## Slide 1
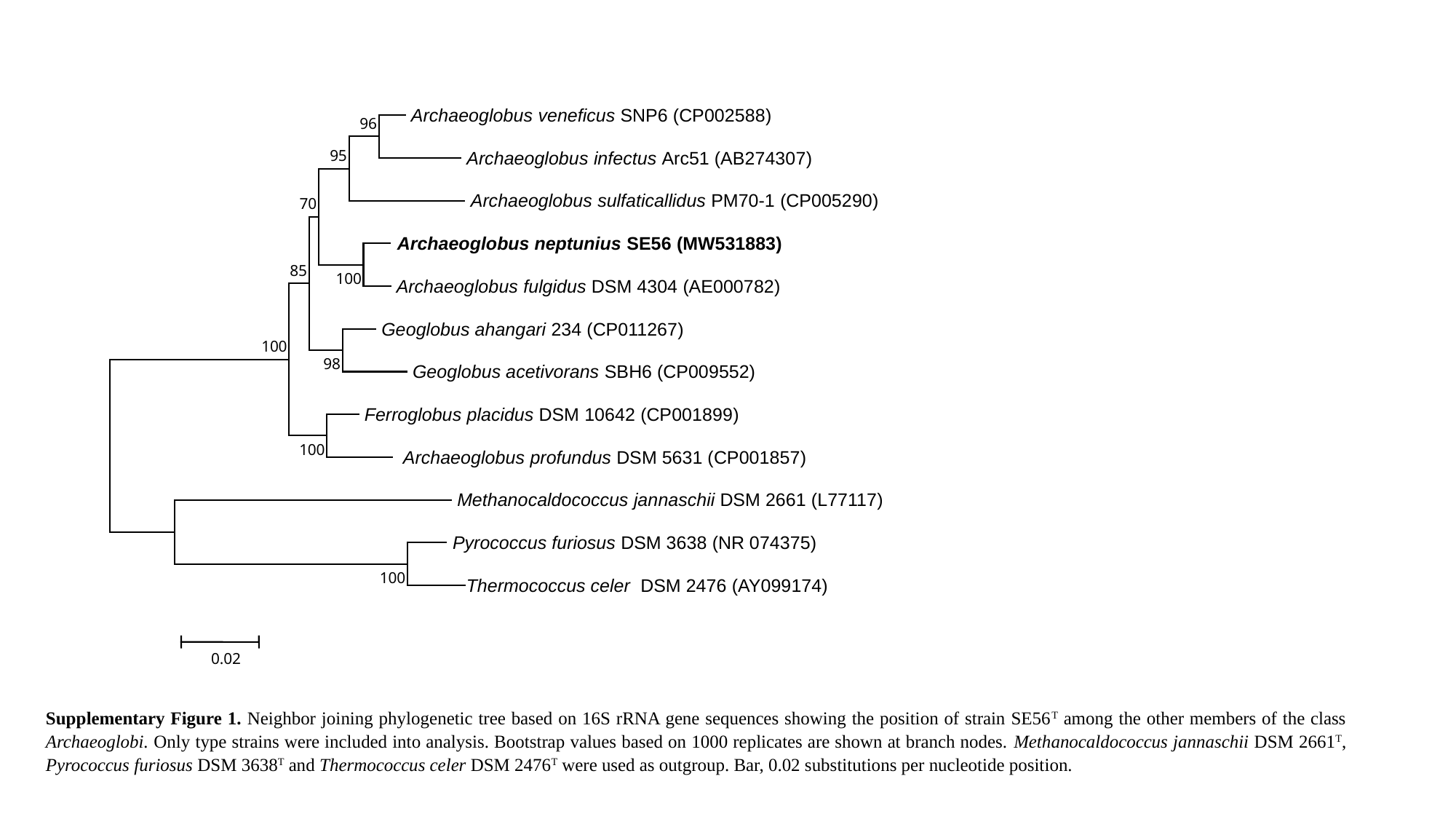

Archaeoglobus veneficus SNP6 (CP002588)
96
95
 Archaeoglobus infectus Arc51 (AB274307)
 Archaeoglobus sulfaticallidus PM70-1 (CP005290)
70
 Archaeoglobus neptunius SE56 (MW531883)
85
100
 Archaeoglobus fulgidus DSM 4304 (AE000782)
 Geoglobus ahangari 234 (CP011267)
100
98
 Geoglobus acetivorans SBH6 (CP009552)
 Ferroglobus placidus DSM 10642 (CP001899)
100
 Archaeoglobus profundus DSM 5631 (CP001857)
 Methanocaldococcus jannaschii DSM 2661 (L77117)
 Pyrococcus furiosus DSM 3638 (NR 074375)
100
Thermococcus celer DSM 2476 (AY099174)
0.02
Supplementary Figure 1. Neighbor joining phylogenetic tree based on 16S rRNA gene sequences showing the position of strain SE56T among the other members of the class Archaeoglobi. Only type strains were included into analysis. Bootstrap values based on 1000 replicates are shown at branch nodes. Methanocaldococcus jannaschii DSM 2661T, Pyrococcus furiosus DSM 3638T and Thermococcus celer DSM 2476T were used as outgroup. Bar, 0.02 substitutions per nucleotide position.

## Slide 2
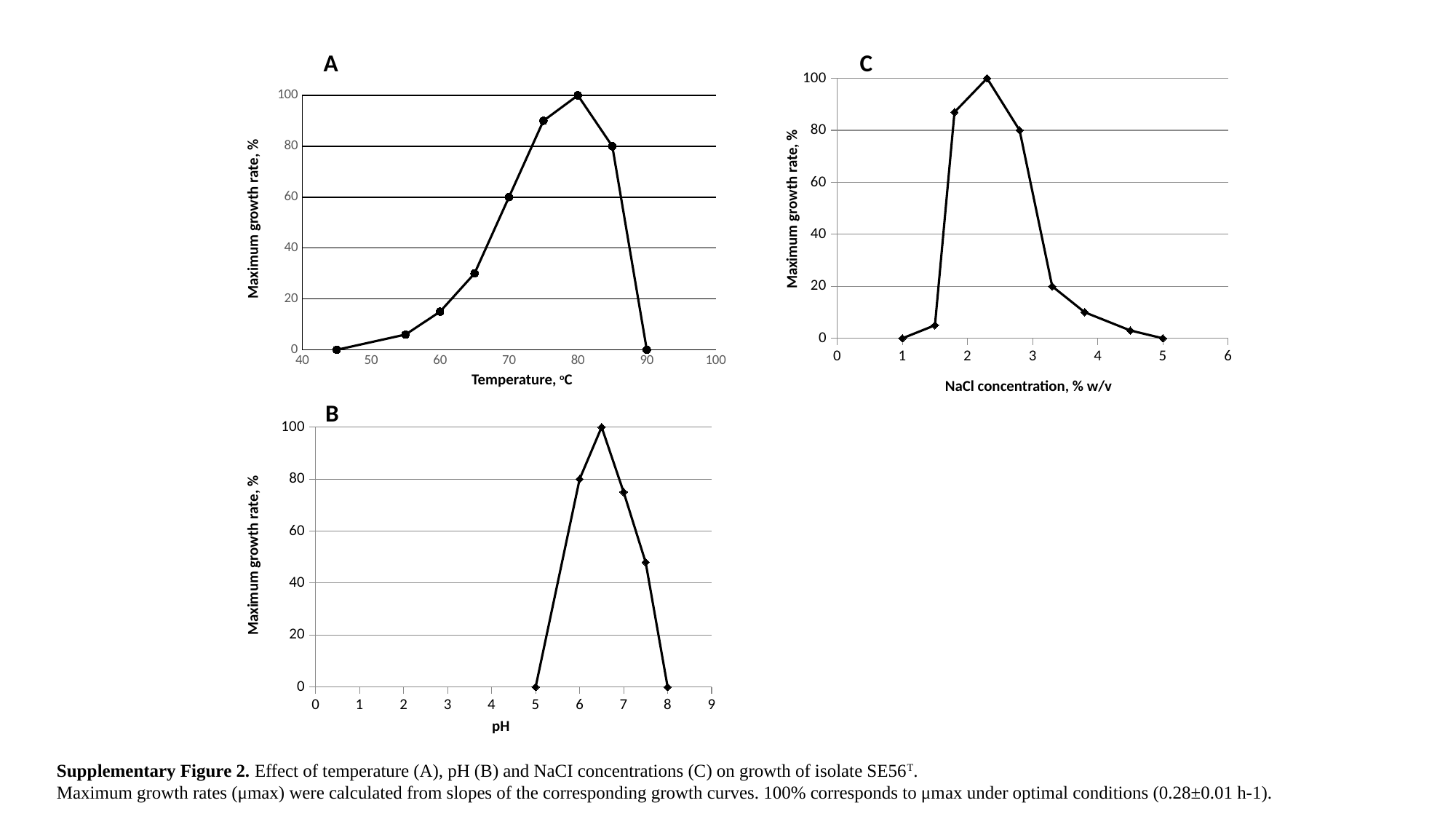

A
C
### Chart
| Category | |
|---|---|Maximum growth rate, %
NaCl concentration, % w/v
### Chart
| Category | % |
|---|---|Maximum growth rate, %
Temperature, oC
B
### Chart
| Category | |
|---|---|Maximum growth rate, %
pH
Supplementary Figure 2. Effect of temperature (A), pH (B) and NaCI concentrations (C) on growth of isolate SE56T.
Maximum growth rates (μmax) were calculated from slopes of the corresponding growth curves. 100% corresponds to μmax under optimal conditions (0.28±0.01 h-1).
