# Supplementary material for: Physiological and Genomic Characterization of a Hyperthermophilic Archaeon Archaeoglobus neptunius sp. nov. Isolated From a Deep-Sea Hydrothermal Vent Warrants the Reclassification of the Genus Archaeoglobus
Source: Front Microbiol. 2021 Jul 16;12:679245. doi: 10.3389/fmicb.2021.679245 (PMC8322695; doi:10.3389/fmicb.2021.679245)
Supplement: Supplementary file 3 [file Table_2.DOCX]

**SupplementaryTable 2.** Pairwise ANI determined for members of the class *Archaeglobi* using MiSI algorithm

|  | *A. neptunius* SE56^T^ | *A. fulgidus*  VC-16^T^ | *A. sulfaticallidus* PM70-1^T^ | *A. profundus* AV18^T^ | *A. veneficus* SNP6^T^ | *G. ahangari* 234^T^ | *G. acetivorans* SBH6^T^ | *F. placidus* DSM 10642^T^ |
| --- | --- | --- | --- | --- | --- | --- | --- | --- |
| *A. neptunius* SE56^T^ | 100 | 74 | 69 | 68 | 69 | 69 | 69 | 69 |
| *A. fulgidus* VC-16^T^ | 74 | 100 | 70 | 69 | 70 | 71 | 70 | 70 |
| *A. sulfaticallidus* PM70-1^T^ | 69 | 70 | 100 | 69 | 70 | 69 | 69 | 69 |
| *A. profundus* AV18^T^ | 68 | 69 | 69 | 100 | 69 | 68 | 68 | 69 |
| *A. veneficus* SNP6^T^ | 69 | 70 | 70 | 69 | 100 | 70 | 69 | 69 |
| *G. ahangari* 234^T^ | 69 | 71 | 70 | 68 | 70 | 100 | 74 | 71 |
| *G. acetivorans* SBH6^T^ | 69 | 70 | 69 | 68 | 69 | 74 | 100 | 70 |
| *F. placidus* DSM 10642^T^ | 69 | 70 | 69 | 69 | 69 | 71 | 70 | 100 |

**Supplementary Table 3**. Alignment fraction (AF) values determined for members of the class *Archaeglobi*

|  | *A. neptunius* SE56^T^ | *A. fulgidus*  VC-16^T^ | *A. sulfaticallidus* PM70-1^T^ | *A. profundus* AV18^T^ | *A. veneficus* SNP6^T^ | *G. ahangari* 234^T^ | *G. acetivorans* SBH6^T^ | *F. placidus* DSM 10642^T^ |
| --- | --- | --- | --- | --- | --- | --- | --- | --- |
| *A. neptunius* SE56^T^ | 1.00 | 0.62 | 0.29 | 0.21 | 0.27 | 0.30 | 0.26 | 0.27 |
| *A. fulgidus* VC-16^T^ | 0.62 | 1.00 | 0.29 | 0.22 | 0.28 | 0.29 | 0.26 | 0.27 |
| *A. sulfaticallidus* PM70-1^T^ | 0.29 | 0.29 | 1.00 | 0.21 | 0.29 | 0.29 | 0.26 | 0.24 |
| *A. profundus* AV18^T^ | 0.21 | 0.22 | 0.21 | 1.00 | 0.27 | 0.20 | 0.21 | 0.22 |
| *A. veneficus* SNP6^T^ | 0.27 | 0.28 | 0.29 | 0.27 | 1.00 | 0.27 | 0.25 | 0.24 |
| *G. ahangari* 234^T^ | 0.30 | 0.29 | 0.29 | 0.20 | 0.27 | 1.00 | 0.63 | 0.39 |
| *G. acetivorans* SBH6^T^ | 0.27 | 0.26 | 0.26 | 0.20 | 0.25 | 0.63 | 1.00 | 0.37 |
| *F. placidus* DSM 10642^T^ | 0.27 | 0.27 | 0.24 | 0.22 | 0.24 | 0.39 | 0.37 | 1.00 |
